# Synthesis of Fe^2+^ Substituted High-Performance LiMn_1−x_Fe_x_PO_4_/C (x = 0, 0.1, 0.2, 0.3, 0.4) Cathode Materials for Lithium-Ion Batteries via Sol-Gel Processes

**DOI:** 10.3390/molecules26247641

**Published:** 2021-12-16

**Authors:** Kaibin Fang, Jihua Zhu, Qian Xie, Yifei Men, Wei Yang, Junpeng Li, Xinwei Yu

**Affiliations:** 1 School of Chemistry and Chemical Engineering, Guangzhou University, Guangzhou 510006, China; kbfang608@gmail.com (K.F.); xieqian018@gmail.com (Q.X.); menyifei2000@163.com (Y.M.); yxw-gzdx@163.com (X.Y.); 2 Zhuhai CosMX Battery Co., Ltd., Zhuhai 519180, China; zhujh125@126.com; 3 School of Chemical Engineering and Technology, Guangdong Industry Polytechnic, Guangzhou 510300, China

**Keywords:** cathode materials, iron doping ratio, LiMn_1−x_Fe_x_PO_4_, sol-gel processes

## Abstract

A series of carbon-coated LiMn_1−x_Fe_x_PO_4_ (x = 0, 0.1, 0.2, 0.3, 0.4) materials are successfully constructed using glucose as carbon sources via sol-gel processes. The morphology of the synthesized material particles are more regular and particle sizes are more homogeneous. The carbon-coated LiMn_0.8_Fe_0.2_PO_4_ material obtains the discharge specific capacity of 152.5 mAh·g^−1^ at 0.1 C rate and its discharge specific capacity reaches 95.7 mAh·g^−1^ at 5 C rate. Iron doping offers a viable way to improve the electronic conductivity and lattice defects of materials, as well as improving transmission kinetics, thereby improving the rate performance and cycle performance of materials, which is an effective method to promote the electrical properties.

## 1. Introduction

Lithium-ion batteries are recognized as ideal energy-storage and output-power sources for new energy vehicles due to their excellent characteristics, such as excellent cycle performance and environmentally friendly characteristics. In the past few years, lithium transition metal phosphate compounds (LiMPO_4_, M = Fe, Mn, Co, Ni) have been considered as an encouraging cathode material for lithium-ion batteries [1,2,3,4,5,6,7,8,9]. Among many phosphate cathode materials, olivine lithium iron phosphate (LiFePO_4_) has been well studied as a cathode material for lithium-ion batteries due to its high theoretical capacity of 170 mAh·g^−1^ and good thermochemical stability. Whereas, LiFePO_4_ has the weaknesses of bad ionics diffusion rate (1.8 × 10^−14^ cm^2^·s^−1^) and poor conductivity (10^−9^–10^−10^ cm^2^·s^−1^); moreover, its working voltage is only 3.5 V (compared with Li^+^/Li), hindering the further wide application of LiFePO_4_ [9,10,11,12,13].

Alternatively, olivine lithium manganese phosphate (LiMnPO_4_) is receiving widespread attention because of its higher operating voltage platform (4.1 V vs. Li^+^/Li) [14,15], which is 20% higher than that of LiFePO_4_, indicating the potential theoretical energy density of LiMnPO_4_ may be 1.2 times that of LiFePO_4_, which is the biggest advantage of LiMnPO_4_ over LiFePO_4_. Similar to LiFePO_4_, LiMnPO_4_ has low ionic (10^−9^–10^−7^ cm^2^·s^−1^) and electronic conductivity (10^−11^–10^−8^ S/cm), and the cost of LiMnPO_4_ is also quite low [7,15,16,17]. The Jahn-Teller distortion exists during the charge-discharge process of LiMnPO_4_, which causes the cycle capacity to decay rapidly, and may also cause side reactions between electrode and electrolyte materials [16].

The reducibility of carbon can inhibit the oxidation of Mn^2+^ and the growth and agglomeration of material particles to a certain extent, mainly by reducing the particle size of the cathode material, preventing the oxidation of Fe^2+^ [17], increasing the specific surface area, and shortening the diffusion path of Li^+^ to promote the material electrochemical performance. Mixing organic carbon source and inorganic carbon source for coating also helps to improve the performance of materials [5]. At the same time, the carbon coating layer can form a transport network between different particles and improve the conductivity of materials [18]. Note that carbon materials with a mass fraction of 25% should be used for coating modification to achieve the purpose of expanding battery capacity [19].

The sol-gel process is to dissolve the complexing agent and stoichiometric amounts of raw materials in a solvent, react in an inert gas atmosphere to form a gel, and then calcinate to obtain the final product. The sol-gel processes have many advantages, such as the low temperature of the reaction, small particle size, and uniform distribution of composition compared to solid-phase reactions [20,21,22,23]. In addition, one of the characteristics the sol-gel process has is to control the particle size of powder easily, which is superior to the solid-phase reaction.

The formation of solid solutions between LiFePO_4_ and LiMnPO_4_ starts a new method in the research and design of electrode materials due to its combination of the high electron transportability of LiFePO_4_ and the high-voltage platform of LiMnPO_4_, which was based on olivine-shaped phosphate material with a potential application in HEV [24]. The doping of Fe ions dilutes the concentration of Mn ions, thereby reducing the Jahn–Teller distortion caused by Mn^3+^ [18]. The introduction of Mn^2+^ in the lattice can widen the lithium-ion diffusion channel to some extent, and the presence of Fe^2+^ can improve electrode kinetics. The interaction between them [25,26] makes LiFe_1-x_Mn_x_PO_4_/C solid solution exhibit excellent electrochemical performances and kinetic properties.

In this paper, Mn(CH_3_COO)_2_·4H_2_O, LiOH·H_2_O, FeC_2_O_4_·2H_2_O, and NH_4_H_2_PO_4_ were used as raw materials to prepare Fe^2+^ substituted LiMn_1−x_Fe_x_PO_4_/C (0 < x ≤ 0.4) positive electrode materials via sol-gel processes. At the same time, we used the same method to synthesize LiMnPO_4_ for comparison with the modified material. LiMn_1-x_Fe_x_PO_4_/C electrode materials’ electrochemical properties and their microstructure were investigated by adjusting the ratio of iron and manganese. Thereafter, samples that had remarkable electrochemical performance were selected comprehensively.

## 2. Result and Discussion

### 2.1. Phase Structure Analysis

**Figure 1:**
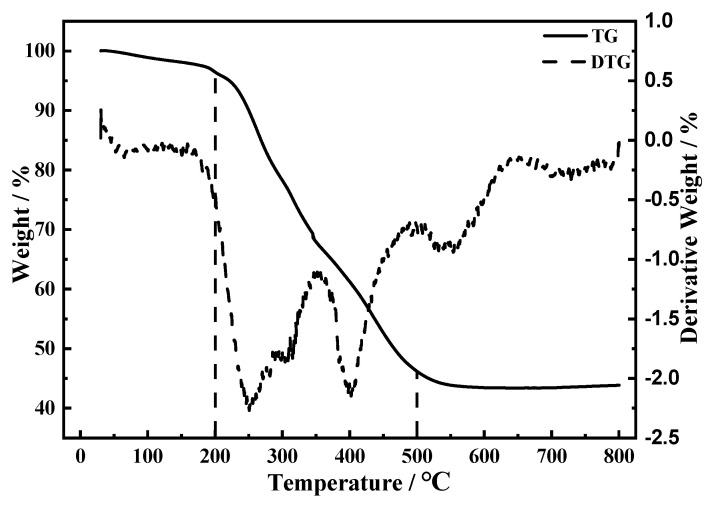
TG-DTG curve of precursor of LiMnPO_4_/C samples synthesized through sol-gel route.

Figure 1 is the thermogravimetric curve of precursor of LiMnPO_4_/C sample synthesized by sol-gel processes. As can be seen from the figure, the differential thermal gravity (DTG) curve shows two strong endothermic peaks at 250 °C and 400 °C; correspondingly, the TG curve has two obvious weight-loss steps in the range of 25 °C to 800 °C. When the temperature range is between 200 °C and 500 °C, the weight loss of the sample is approximately 50%, which corresponds to the high temperature decomposition process of the precursor organic matter, where the mass loss in the range of 200 °C to 300 °C corresponds mainly to the decomposition of NH_4_H_2_PO_4_ and citric acid, and the weight loss in the range of 300 °C to 500 °C corresponds to the decomposition of Mn(CH_3_COO)_2_, accompanied by the generation of LiMnPO_4_. When the temperature is higher than 500 °C, the TG curve tends to be basically flat and enters the relatively constant weight region, indicating that there is no more phase change occurring at temperatures higher than 500 °C, and LiMnPO_4_ has crystallized completely, and that higher temperature treatment only increases the crystallinity of LiMnPO_4_. As can be seen from the TG-DTG curve, the decomposition reactions of the precursors and the chemical reactions that take place between them occur mainly before 500 °C, while the weight loss between 30 °C and 200 °C is only 2%, which corresponds to the evaporation process of water contained in the precursors. When the temperature is between 600–800 °C, the weight loss rate is 1%. Therefore, the two-stage sintering method is adopted in the whole sintering process. See the experiment operation section for details.

**Figure 2:**
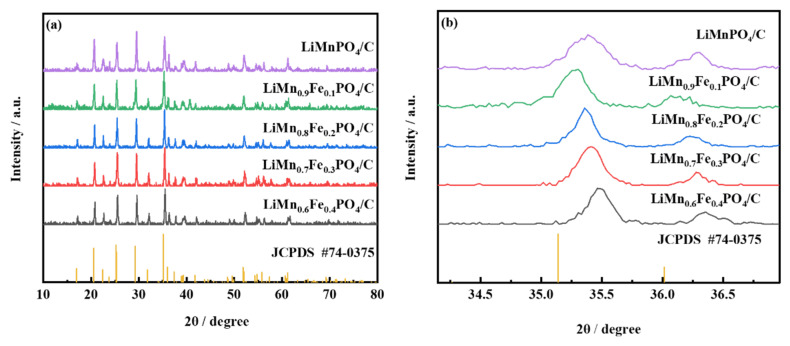
XRD patterns of LiMn_1−x_Fe_x_PO_4_/C (0 ≤ x ≤ 0.4) samples; (**a**) XRD data for LiMn_1−x_Fe_x_PO_4_ (0 ≤ x ≤ 0.4) samples, (**b**) peak shift of XRD patterns.

Figure 2 shows XRD patterns of LiMn_1−x_Fe_x_PO_4_/C (0 ≤ x ≤ 0.4) synthesized by the sol-gel processes. Compared with the standard card (JCPDS NO.74-0375), the positions of the diffraction peaks of each sample were highly consistent with the standard card, so the materials had an olivine-type LiMnPO_4_ structure with Pnmb space group. This indicated that Fe-doping did not cause significant changes in LiMnPO_4_ structure and crystal form. Hence it suggested that Fe^2+^ had successfully entered the crystal space. No obvious carbon peak was observed in the composite material spectrum, illustrating the amorphous nature of the residual carbon layer and its low content [27,28]. As shown in Figure 2b, the diffraction peaks of each sample shifted slightly toward the increase of the diffraction angle with the increase of the Fe-doped amount. This is because Fe^2+^ partially replaces the Mn^2+^ site and the Fe ion radius (0.92 Å) is smaller than the Mn ion radius (0.97 Å); the position of the diffraction peak had slightly shifted [10]. Table S1 (Supplementary Materials) shows the carbon content of LiMn_1−x_Fe_x_PO_4_/C (0 ≤ x ≤ 0.4) materials. As can be seen from Table S1 (Supplementary Materials), the carbon content of different samples was about 7.2%, and the carbon content of LiMn_1−x_Fe_x_PO_4_/C materials was similar.

For LiMn_1−x_Fe_x_PO_4_/C (0 ≤ x ≤ 0.4) cathode materials, their lattice parameters after Rietveld refinement are summarized in Table 1. The calculated lattice parameters decrease linearly along with the shrink of the unit cell with the increase of the Fe-doping amount, and the change is approximately linear, which was consistent with Vegard’s law. This indicated that Fe^2+^ doping could cause the lattice shrinkage of LiMnPO_4_, but the crystal structure of LiMnPO_4_ was not affected [26,29]. Thus, it can be seen that Fe^2+^ was successfully embedded in the olivine structure of LiMnPO_4_.

### 2.2. Micromorphology Analysis

**Figure 3:**
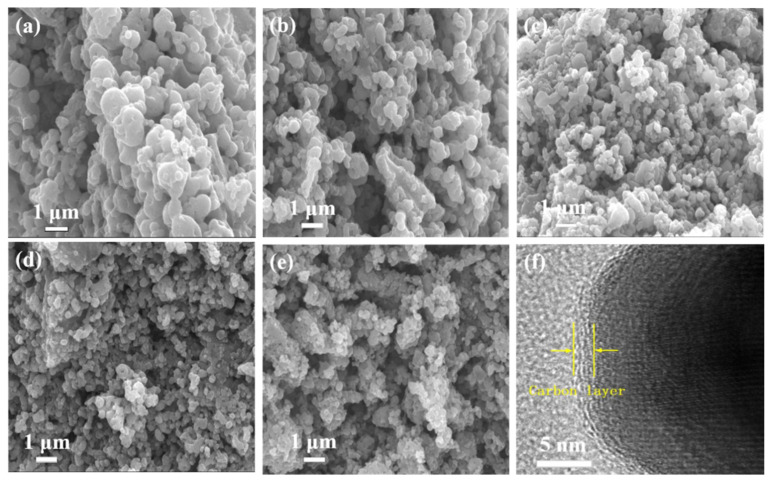
SEM images of (**a**) LiMnPO_4_/C, (**b**) LiMn_0.9_Fe_0.1_PO_4_/C, (**c**) LiMn_0.8_Fe_0.2_PO_4_/C, (**d**) LiMn_0.7_Fe_0.3_PO_4_/C, (**e**) LiMn_0.6_Fe_0.4_PO_4_/C, and HRTEM image of (**f**) LiMn_0.8_Fe_0.2_PO_4_/C.

The morphology of LiMn_1−x_Fe_x_PO_4_/C (0 ≤ x ≤ 0.4) materials is presented in Figure 3. It can be seen from Figure 3a–e that the distribution of particle sizes for LiMn_1−x_Fe_x_PO_4_/C materials are very uniform. The primary particle size is about 300 nm. Synthesized materials had a relatively regular morphology, and most of them are spherical. This means that iron doping helps to improve the morphology of the material particles. Figure 3f shows an HRTEM image of the LiMn_0.8_Fe_0.2_PO_4_/C cathode material, from which it can be clearly seen that an amorphous carbon coating with a thickness of approximately 1.5 nm is present on the surface of the material. The particle size distribution statistics shown in Figure S1 (Supplementary Materials) verified that the LiMn_0.8_Fe_0.2_PO_4_/C cathode material possesses a more uniform particle size distribution than the other four samples, with an average particle size of 260 nm. The particle size of materials was decreased first and then increased with the increase of the amount of Fe-doping, and agglomeration of spherical particles intensified. Increasing the particle size of materials will increase the migration distance of lithium ions during charging and discharging. Thus, the utilization rate of active substances is reduced [22], which is not conducive to modify the electrochemical performance of materials.

### 2.3. Electrochemical Analysis

**Figure 4:**
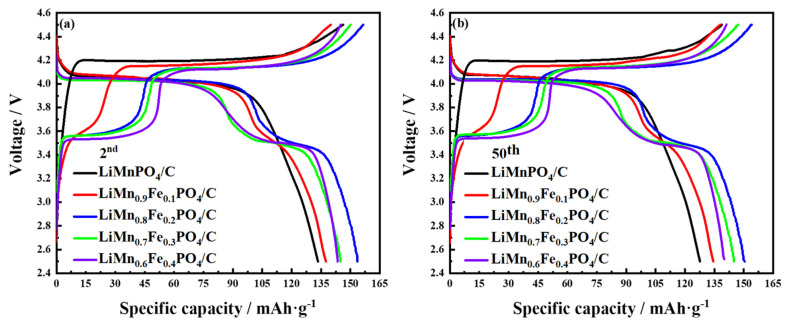
Charge-discharge profiles of LiMn_1-x_Fe_x_PO_4_/C (0 ≤ x ≤ 0.4) synthesized through sol-gel route; (**a**) the second charge/discharge profiles of LiMn_1-x_Fe_x_PO_4_/C (0 ≤ x ≤ 0.4) samples at 0.1 C; (**b**) charge/discharge profiles of LiMn_1−x_Fe_x_PO_4_/C (0 ≤ x ≤ 0.4) samples after 50 cycles at 0.1 C.

The electrochemical performance of materials was tested under constant current charging and discharging mode. Figure 4 is the charge–discharge capacity diagram of LiMn_1−x_Fe_x_PO_4_/C (0 ≤ x ≤ 0.4) cathode materials at a rate of 0.1 C. Theoretically, since the total capacity of each synthetic sample is identical during the charge–discharge process, the lengths of charge/discharge platforms should be equal. However, since the maximum overpotential causes the minimum phase-change activation energy, the length of charge/discharge platforms in the 3.5 V and 4.1 V regions is not symmetrical [30]. It can be seen from Figure 4 that LiMn_1−x_Fe_x_PO_4_/C materials exhibit charge/discharge voltage plateaus at 3.5 V, corresponding to the Fe^2+^/Fe^3+^ redox couple, and at 4.1 V, associating with the Mn^2+^/Mn^3+^ redox couple, of which the LiMn_0.8_Fe_0.2_PO_4_/C material had the widest charge/discharge platform. The high operating voltage platform is an important parameter for the cathode material to meet the high energy-density requirement of electronic products. The discharge specific capacity of the LiMn_0.8_Fe_0.2_PO_4_/C material also reached a peak of 152.5 mAh·g^−1^. Therefore, the LiMn_0.8_Fe_0.2_PO_4_/C material had the smallest degree of polarization during charging and discharging among several prepared materials, and had the highest specific discharge capacity and battery energy density [26]. At the same time, it was confirmed that the Mn site was successfully partially replaced by Fe, and Fe substitution had a positive effect on the two-phase transition between the charge/discharge reactions.

**Figure 5:**
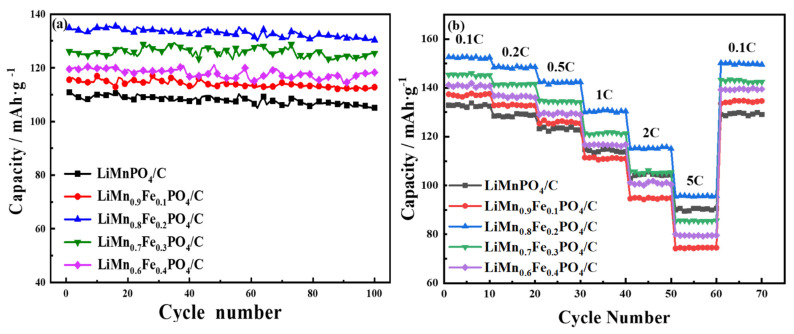
(**a**) Cycle performance at 1 C and (**b**) rate capability of LiMn_1−x_Fe_x_PO_4_/C (0 ≤ x ≤ 0.4) samples.

The partial substitution of Fe at Mn sites can significantly enhance the lithium-ion diffusion and reduce the polarization of LiMnPO_4_, which helps to suppress the Jahn–Teller distortion. With the increase of Mn content and the amount of iron substitution decreases in olivine materials, the discharge specific capacity of materials decreases instead during the charge/discharge cycle. This is due to the loss of the electrochemical activity of the Mn^2+^/Mn^3+^ redox center, which has been reported in the previous literature [30,31]. LiMn_1−x_Fe_x_PO_4_/C materials had better charge–discharge performance, and the cycle performance was enhanced, as shown in Figure 5a. These voltage plateaus agree well with the anode and cathode peaks in the CV curves and are also consistent with the particle size variation trend of LiMn_1−x_Fe_x_PO_4_/C materials (Figure S1, Supplementary Materials).

Figure 5 showed the cycle performance of LiMn_1−x_Fe_x_PO_4_/C (0 ≤ x ≤ 0.4) cathode materials and rate performance at current densities of 0.1 C, 0.2 C, 0.5 C, 1 C, 2 C, and 5 C, respectively. The high rate performance at high operating voltage should be attributed to the enhancement of lithium-ion diffusion kinetics and the better contact between the cathode material and the current collector due to the uniform coating of conductive carbon material [32]. As can be seen from Figure 5b, we can see that the discharge specific capacity of LiMn_1-x_Fe_x_PO_4_/C materials decreases with the increase of rate. When the iron-manganese ratio was 2:8, the discharge specific capacities of materials under the condition of 0.1 C to 5 C were higher than that of the other four synthetic materials. The discharge specific capacity at 5 C rate is only 95.7 mAh·g^−1^, which was 73.3% of 1 C rate and 56.3% of the theoretical capacity, illustrating that the material had a longer charge/discharge voltage platform and a higher discharge specific capacity. Therefore, LiMn_0.8_Fe_0.2_PO_4_/C exhibited excellent rate performance and cycling stability.

The electrochemical properties of the composite material largely depended on the ratio of Fe to Mn [2]. Increasing the proportion of manganese may cause the discharge specific capacity of the material to decrease. Due to the intense Jahn-Teller distortion caused by Mn^3+^, the lithium-ion extraction process inside the LiMnPO_4_ material is hindered, and the LiMnPO_4_ material has an olivine structure, and the structural disorder in the crystal lattice may hinder lithium exchange in the [010] channel to a certain extent [33]. The mixed arrangement of Mn^2+^ at the Li^+^ position hinders the transmission of lithium ions in one-dimensional channels and reduces the electrochemical activity of the material [29,34], while the addition of iron ions increases the capacity of the electrode material, which means that iron doping is more beneficial to the deintercalation/intercalation process of Li^+^ [1,35]. At the same time, the Jahn-Teller distortion is related to the Mn-Mn distance in the crystal structure [34,35,36]. It is presumed that the ion radius of Fe^2+^ (0.92 Å) is smaller than the ion radius of Mn^2+^ (0.97 Å), so substitution with Fe^2+^ will increase the Mn-Mn distance and reduced the Jahn-Teller distortion. When Fe is embedded in the position of Mn in the crystal lattice of LiMnPO_4_, the smaller Fe atom energy will leave more space for the migration of Li^+^ due to the small size of the Fe atom, and inhibit the dissolution of Mn^3+^ in the electrolyte, thus stabilizing the crystal ion configuration of materials. Iron-doping modification can improve the ionic conductivity and lattice defects of materials to a certain extent, and enhance its transmission kinetics, thereby improving the rate performance and cycle performance of materials under charge/discharge conditions, which is an effective improvement method for electrochemical performance.

**Figure 6:**
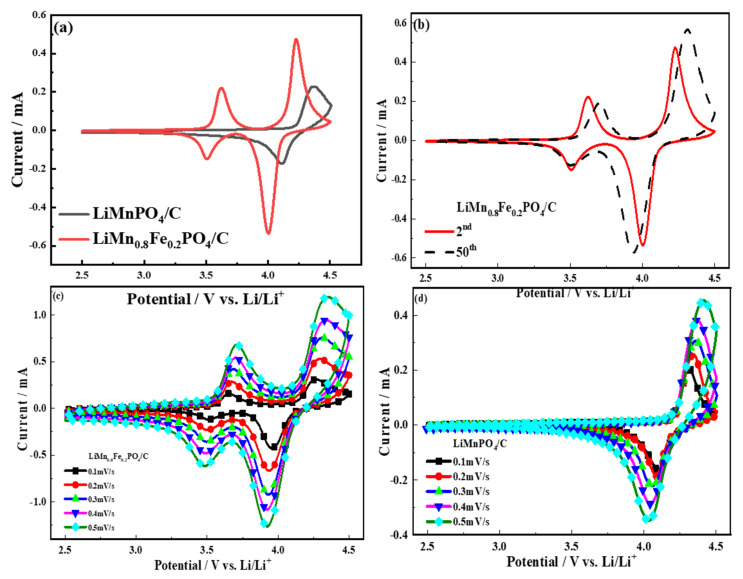
CV curves of LiMn_0.8_Fe_0.2_PO_4_/C sample; (**a**) cyclic voltammogram of LiMn_0.8_Fe_0.2_PO_4_ /C sample and LiMnPO_4_/C sample at the scan rate of 0.1 mV/s; (**b**) cyclic voltammograms of the second and fiftieth circles of the LiMn_0.8_Fe_0.2_PO_4_/C sample; (**c**) cyclic voltammograms of LiMn_0.8_Fe_0.2_PO_4_/C samples at different scan rates; (**d**) cyclic voltammograms of LiMnPO_4_/C samples at different scan rates.

Figure 6 shows the cyclic voltammetry curves of the LiMn_0.8_Fe_0.2_PO_4_/C cathode material and the LiMnPO_4_/C sample. The oxidation peak and reduction peak of the two samples have good symmetry. The CV curves of the LiMn_0.8_Fe_0.2_PO_4_/C cathode material show two pairs of peaks, one at 3.51/3.62 V, corresponding to Fe^2+^/Fe^3+^ redox couple, and the other at 4.0/4.23 V, associating with Mn^2+^/Mn^3+^ redox couple. The LiMnPO_4_/C material showed only one pair of redox couple associated with Mn^2+^/Mn^3+^, as shown in Figure 6d. The anodic peak current was equal to the corresponding cathodic peak current, and the redox peak shape was not destroyed, which indicated that the prepared sample had good reversibility. Two of the materials had good cycle stability, and the ratio of the peak area corresponded to the stoichiometric ratio of Mn/Fe. The CV curve showed that the potential difference of the LiMn_0.8_Fe_0.2_PO_4_/C cathode material between oxidative and reductive peaks was smaller than the LiMnPO_4_/C sample, and the degree of polarization was lower than the LiMnPO_4_/C sample. The energy density of the modified material is linked to the redox potential of transition metal ions [36]. The results showed that iron doping increases the diffusion coefficient of lithium ions, and especially accelerates the conversion between Mn^2+^↔Mn^3+^; the peak in the CV test proved the above statement. The oxidation and reduction peaks of the Mn^2+^/Mn^3+^ and Fe^2+^/Fe^3+^ redox pairs were sharper, and the peak intensity of Fe^2+^/Fe^3+^ increased gradually. This phenomenon can be attributed to the fact that as the iron content in the material increases, the intercalation/deintercalation kinetics of lithium occurring at the electrode/electrolyte interface and the diffusion rate of lithium ions in the crystal lattice also increase.

The CV curves showed that the cathodic peak current was equal to the corresponding highest anodic peak, which indicated that the prepared sample had good chemical reversibility. With the change of the scanning rate, the oxidation and reduction peaks shape was not destroyed, and the potential difference between the oxidation voltage and the reduction voltage changed little. Nanoparticles can ensure that the material has a high lithium-ion diffusion coefficient. For the reversible reaction, the Randles-Sevcik equation can be used to calculate the chemical diffusion coefficient of lithium ions using the peak current during scanning at different sweep speeds:

(1) $$I_{p}=2.69\times 10^{5}ACD_{{\mathrm{Li}}^{+}}^{\frac{1}{2}}n^{\frac{3}{2}}v^{\frac{1}{2}}\left(25\;℃\right)$$

**Figure 7:**
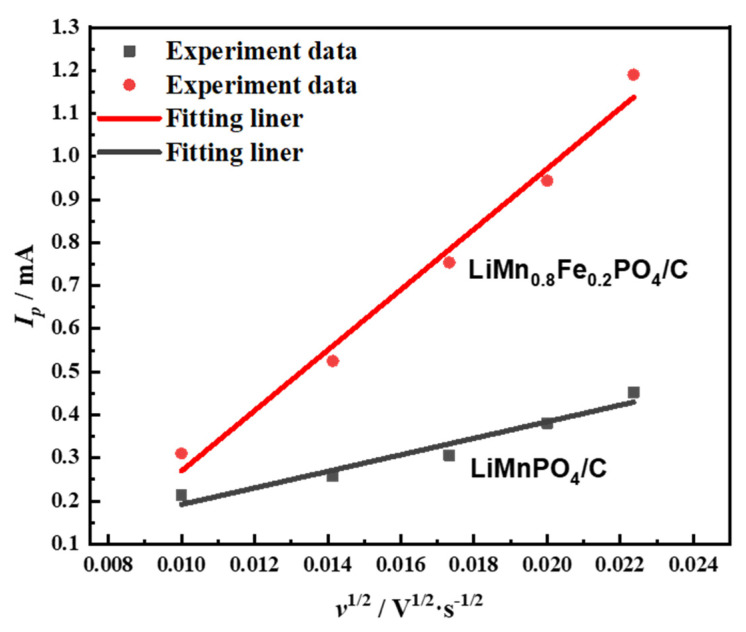
Linear response of the cathodic peak current (I_p_) as a function of square root of the scan rate (ν).

*I_p_* is the peak current of the positive electrode at different scanning speeds, *ν* is the scan rate (V·s^−1^), *A* is the area of the electrode pole piece (cm^2^), *C*_Li_ is the lithium-ion concentration, *D*_Li_ is the lithium-ion diffusion coefficient (cm^2^·s^−1^), and *n* is the number of electrons in the redox process. The linear response of the cathodic peak current (*Ip*) as a function of the square root of the scan rate (*ν*) is shown in Figure 7.

**Figure 8:**
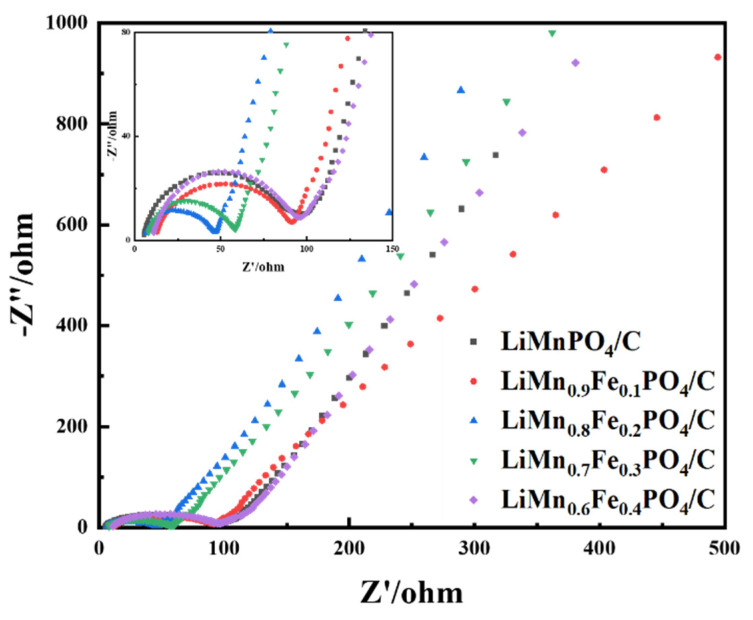
EIS plots of LiMn_1−x_Fe_x_PO_4_ /C (0 ≤ x ≤ 0.4) samples.

The electrochemical impendence spectroscopy spectrum test was carried out to further analyze the electrochemical kinetics of the above samples. The EIS spectrum consists of a high-frequency intercept, a compressed semicircle from high to intermediate frequencies, and a low-frequency oblique line. The small intercept at a high frequency corresponds to the solution resistance of the battery and the recessed semicircle at the high-frequency-intermediate frequency are related to the charge transfer resistance at the electrode/electrolyte interface and the electric double layer capacitance between the electrolyte and the positive electrode. The oblique line is attributed to the Warburg impedance corresponding to lithium-ion diffusion in the electrode. From Figure 8, LiMn_1−x_Fe_x_PO_4_/C materials showed small interface resistance and faster diffusion of Li^+^, which could be ascribed to the doping of Fe. A smaller R_ct_ value is conducive to rapid electrochemical reactions and can improve the material’s electrochemical performance. This result was also in agreement with the results of Figure 4 and Figure 5. Through comparison, it was found that the EIS impedance value of samples were maintained between 40–100 Ω, and the charge transfer resistance values were reduced by metal ion doping and carbon coating. Among them, the LiMn_0.8_Fe_0.2_PO_4_/C sample had the smallest impedance, indicating that iron doping significantly improved the electronic conductivity of the crystal of the LiMnPO_4_ sample, and the EIS results were consistent with the electrochemical performance of LiMn_1−x_Fe_x_PO_4_/C samples.

## 3. Experimental Section

### 3.1. Material and Methods

LiMn_1-x_Fe_x_PO_4_/C (x = 0, 0.1, 0.2, 0.3, 0.4) composites were synthesized via a sol-gel process. LiOH·H_2_O (99.0%, Macklin, Shanghai, China), Mn(CH_3_COO)_2_ (99.0%, Macklin, Shanghai, China), FeC_2_O_4_ (99.0%, Macklin, Shanghai, China), NH_4_H_2_PO_4_ (99.0%, Damao, Tianjin, China) and 0.125 mol of citric acid (99.8%, Macklin, Shanghai, China) were dissolved in 66.7 mL deionized water, respectively, which ensured the molar ratio of Li:Mn:Fe:P = 1:1 − x:x:1 (x = 0, 0.1, 0.2, 0.3, 0.4). The sols were formed after 6 h reaction with 80 °C constant-temperature water bath, and then the excess water was removed by vacuum distillation to get gel. To make the gel fully dried, it was transferred to the vacuum drying box for drying at 140 °C for 24 h. The dried gel mixing with anhydrous glucose (25 wt%) was placed in quartz boats. N_2_, as a protective gas, was introduced into the tube furnace. The mixture was heated from room temperature to 350 °C and kept for 4 h to fully decompose the organic matter; then, the samples were fully crystallized by heating from 350 °C to 600 °C and holding for 10 h. The heating rate of the whole sintering process was set at 3 °С·min^–1^. The calcined product was moved out and ground again, whereafter it was transferred to a dry place for storage.

### 3.2. Characterization of Synthesized Materials

Thermal gravimetric analysis (TG) was accomplished via a PerkinElmer TGA4000 instrument (PerkinElmer, Waltham, MA, USA) at a heating rate of 10 °С·min^–1^ in high purity inert gas to determine the thermal properties of LiMnPO_4_/C precursors, and the temperature range was 30–800 °C. The crystal structure was researched by X-ray diffraction (XRD, PANalytical Xpert Pro, Almelo, The Netherlands) with the Cu Kα source as the incident beam (λ = 1.5406 Å) at a voltage of 40 kV and a current of 10 mA, and scanning the 2θ range from 5 to 80° at a scan rate of 10°·min^−1^. The carbon content of LiMn_1−x_Fe_x_PO_4_/C materials was tested using elemental analyzer (Vario EL III, Elementar, Berlin, Germany). JADE refined the XRD spectrum to estimate the lattice parameters. The morphology of synthetic materials was observed by scanning electron microscopy (SEM, JSM-7001F, JEOL, Tokyo, Japan), one of the most commonly used surface topography observation methods. The High Resolution Transmission Electron Microscope (HRTEM) image of carbon coating was observed with transmission electron microscopy (TEM, JEM 2100F, JEOL, Tokyo, Japan).

### 3.3. Electrode Preparation and Electrochemical Characterization

The tested electrodes of LiMn_1−x_Fe_x_PO_4_/C (0 ≤ x ≤ 0.4) were made by mixing 80 wt% active material, 10 wt% acetylene black, and 10 wt% polyvinylidene fluoride (PVDF) into a slurry substance. After adding *N*-methyl-2-pyrrolidone (NMP) as a solvent to the mixed materials, the mixed slurry was coated onto an aluminum-foil current collector to prepare electrode materials. The material loading of the cathode film is about 1.6–2.2 mg·cm^−2^. The electrodes attached to the aluminum foil were cut into 12 mm diameter discs after a roller pressing operation. CR2032 coin cells, which were used to conduct the electrochemical measurements of synthesized composites, were being assembled in an argon-filled glove box. An amount of 1 M LiPF_6_ was selected as the electrolyte, which was dissolved in a mixed solution composed of ethylene carbonate (EC), dimethyl carbonate (DMC), and ethyl methyl carbonate (EMC). The volume ratio of mixed solution is 1:1:1. The rate performance and cycling performance of LiMn_1-x_Fe_x_PO_4_/C (x = 0, 0.1, 0.2, 0.3, 0.4) electrode materials were tested by the Neware battery test system (Neware BTS-5 V-10 mA, Neware, Shenzhen, China) at the potential window between 2.5 and 4.5 V. Cyclic voltammetry (CV) was carried out on the CHI600E electrochemical workstation (CH Instruments, Austin, TX, USA) under the same range of potential window. The electrochemical impendence spectroscopy test (EIS) was conducted using the CHI600E electrochemical workstation with a frequency range of 10^−2^ to 10^5^ Hz and a range of 5 mV.

## 4. Conclusions

In short, we successfully developed a series of LiMn_1−x_Fe_x_PO_4_ /C (0 ≤ x ≤ 0.4) materials with uniform particle size distribution and excellent electrochemical performance through the sol-gel process. By modifying the iron-doping ratio of LiMn_1−x_Fe_x_PO_4_/C materials, it was observed by XRD and SEM that when the iron-manganese ratio was 2:8, the sample had an olivine-type LiMnPO_4_ structure with Pnmb space group. With the increase of Fe doping amount, the calculated lattice parameters decreased, indicating the successful integration of Fe^2+^. When the discharge rate was 0.1 C, the discharge specific capacity was 152.2 mAh·g^−1^, and 130.5 mAh·g^−1^ at a rate of 1 C. The improved properties of modified materials were attributed to the successful insertion of Fe^2+^, so iron doping was an effective method to improve the electrochemical properties. Therefore, LiMn_1−x_Fe_x_PO_4_/C materials are promising cathode materials for lithium-ion batteries.

## Figures and Tables

**Table 1:** Refined lattice parameters of LiMn_1−x_Fe_x_PO_4_/C composites.

Sample	a (Å)	b (Å)	c (Å)	v (Å)^3^
LiMnPO_4_/C	10.445	6.102	4.745	302.61
LiMn_0.9_Fe_0.1_PO_4_/C	10.434	6.088	4.741	301.22
LiMn_0.8_Fe_0.2_PO_4_/C	10.425	6.081	4.737	300. 7
LiMn_0.7_Fe_0.3_PO_4_/C	10.412	6.073	4.732	299.12
LiMn_0.6_Fe_0.4_PO_4_/C	10.405	6.065	4.727	298.14

## Data Availability

Not applicable.

## Supplementary Materials

The following are available. Figure S1: The statistics of particle size distribution of LiMn_1-x_Fe_x_PO_4_/C (0 ≤ x ≤ 0.4) samples; (**a**) LiMnPO_4_/C (based on SEM image(**a**)); (**b**) LiMn_0.9_Fe_0.1_PO_4_/C (based on SEM image(**b**)); (**c**) LiMn_0.8_Fe_0.2_PO_4_/C (based on SEM image(**c**)); (**d**) LiMn_0.7_Fe_0.3_PO_4_/C (based on SEM image(**d**)); (**e**) LiMn_0.6_Fe_0.4_PO_4_/C(based on SEM image(**e**)). Table S1: C contents of the LiMn_1−x_Fe_x_PO_4_/C.

## Sample Availability

Samples of the compounds are not available from the authors.
