# Supplementary material for: Synthesis of Fe2+ Substituted High-Performance LiMn1−xFexPO4/C (x = 0, 0.1, 0.2, 0.3, 0.4) Cathode Materials for Lithium-Ion Batteries via Sol-Gel Processes
Source: Molecules. 2021 Dec 16;26(24):7641. doi: 10.3390/molecules26247641 (PMC8704643; doi:10.3390/molecules26247641)
Supplement: Supplementary file 1 [file molecules-26-07641-s001.zip › molecules-1441765-supplementary.pdf]

Supplementary Materials

Synthesis of Fe^{2+} Substituted High-Performance $LiMn_{1-x}Fe_xPO_4/C$ (x = 0, 0.1, 0.2, 0.3, 0.4) Cathode Materials for Lithium-Ion Batteries via Sol-Gel Processes

Kaibin Fang [1], Jihua Zhu [2], Qian Xie [1], Yifei Men [1], Wei Yang [1,*], Junpeng Li [3,*] and Xinwei Yu [1]

[1] School of Chemistry and Chemical Engineering, Guangzhou University, Guangzhou 510006, China; kbfang608@gmail.com (K.F.); xieqian018@gmail.com (Q.X.); menyifei2000@163.com (Y.M.); yxw-gzdx@163.com (X.Y.)

[2] Zhuhai CosMX Battery Co., Ltd., Zhuhai 519180, China; zhujh125@126.com

[3] School of Chemical Engineering and Technology, Guangdong Industry Polytechnic, Guangzhou 510300, China

* Correspondence: wyang@gzhu.edu.cn (W.Y.); 2006112030@gdip.edu.cn (J.L.); Tel: +86-13719030050; +86-15918790616 (J.L.)

Table S1. C contents of the $LiMn_{1-x}Fe_xPO_4/C$.

Sample	C wt%
$LiMnPO_4/C$	7.23
$LiMn_{0.9}Fe_{0.1}PO_4/C$	7.21
$LiMn_{0.8}Fe_{0.2}PO_4/C$	7.16
$LiMn_{0.7}Fe_{0.3}PO_4/C$	7.28
$LiMn_{0.6}Fe_{0.4}PO_4/C$	7.24

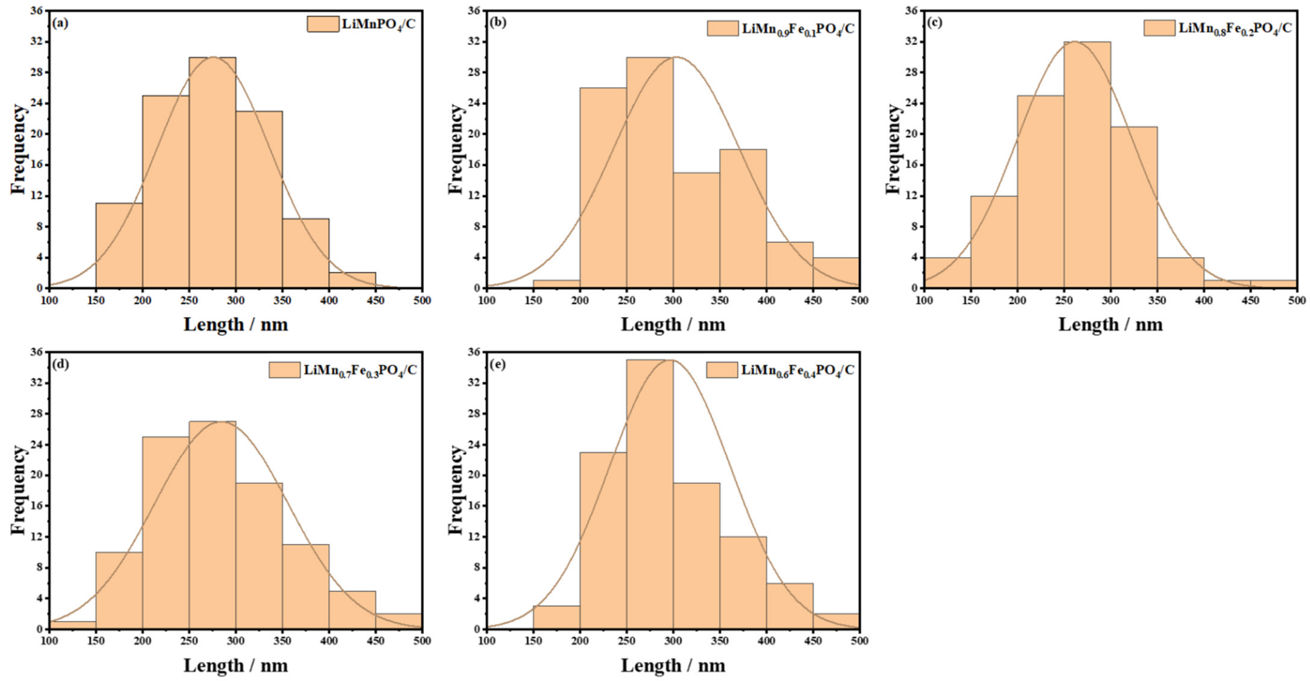

Figure S1. The statistics of particle size distribution of $LiMn_{1-x}Fe_xPO_4/C$ ($0 \leq x \leq 0.4$) samples; (**a**) $LiMnPO_4/C$ (based on SEM image (**a**)); (**b**) $LiMn_{0.9}Fe_{0.1}PO_4/C$ (based on SEM image (**b**)); (**c**) $LiMn_{0.8}Fe_{0.2}PO_4/C$ (based on SEM image (**c**)); (**d**) $LiMn_{0.7}Fe_{0.3}PO_4/C$ (based on SEM image (**d**)); (**e**) $LiMn_{0.6}Fe_{0.4}PO_4/C$ (based on SEM image (**e**)).
